# Supplementary material for: Perception regarding live kidney donation in the general population of South Korea
Source: PLoS One. 2022 Aug 4;17(8):e0272495. doi: 10.1371/journal.pone.0272495 (PMC9352025; doi:10.1371/journal.pone.0272495)
Supplement: S1 File — (DOCX) [file pone.0272495.s001.docx]

**Supporting Information**

**S1 File. All questionnaires conducted in this study**

***Question for selection***

Q1. Where do you live?

① Seoul Metropolitan City ② Metropolitan City ③ Gyeonggi-do ④ Gangwon-do

⑤ Chungcheongbuk-do ⑥ Chungcheongnam-do ⑦ Jeollabuk-do ⑧ Jeollanam-do

⑨ Gyeongsangbuk-do ⑩ Gyeongsangnam-do ⑪ Jeju-do

Q1-1. Which of the following areas do you live in?

① Metropolitan ② Small and medium-sized city (~dong) ③ Town (~gun)

Q2. What is your gender?

① Male ② Female

Q3. How old are you?

____ years old

Q4. What is your final academic background? Please respond based on graduation.

① Seodang/Hanhak ② Literacy ③ Elementary school ④ Middle school

⑤ High school ⑥ 2/3-year university ⑦ 4/6-year university ⑧ Graduate school

***Awareness and perceptions of live donor kidney transplantation***

Q1. Have you ever heard about the live donor kidney transplantation?

① Yes → Go to 1-1

② No → Read the presentation and go to Q2

Q1-1. If you have heard of it, how well do you know?

① I know very well

② I know to some extent

③ I don't know well

④ I only heard it, but I have no idea

| **[Statement]**  Living kidney donation is the act of separating one of the two kidneys for a patient who needs dialysis. |
| --- |

Q2. Do you think a nephrectomy for living kidney donation is safe? Do you feel unsafe?

① Very safe

② It is a little safe

③ Normal

④ It’s not very safe

⑤ It’s not safe at all

Q3. How much do you think living kidney donation will affect your health in the long run? Do you think it won’t affect you?

① It will have a great impact

② It will have a little effect

③ Normal

④ It won't affect you very much

⑤ It will have no effect at all

***Willingness to donate kidney***

Q4. Are you willing to donate your kidneys to a family member, relative, or other person?

① Willing to donate → Go to Q4-1

② I do not intend to donate → Go to Q4-3

⑨ I don't know → Go to Q5

Q4-1. If you donate your kidney, what relationship can you donate? Please rank up to 5th place. If you can donate only to the relationship that is ranked 1^st^, you only need to check the 1^st^ place. As another example, if donation are only available to parents and spouses, you need to rank up to the 2^nd^ priority.

① Parent

② Spouse

③ Brother/Sister

④ Son and daughter

⑤ Relatives within 4 villages

⑥ Relatives with 5 or more connections

⑦ Close friends and other relationships

⑧ others

Q4-2. Why are you willing to donate a kidney? Please answer all applicable reasons.

① For the recipient’s health

② For religious reasons

③ Saving a life feels rewarding

④ After donation, I thought that I could get any financial help from the recipient

⑤ I am convinced that there would be no other physical problems even after a kidney transplant

⑥ Others

→ After answering, go to Q5

Q4-3. If you are not willing to donate your kidneys, why? Please answer all applicable reasons.

① Because of vague fear due to lack of information

② Because it is difficult to access actual cases of kidney transplantation

③ Because of fear of possible medical complications after donation

④ Because fear of effects on the kidneys and other organs after nephrectomy

⑤ Because of fear of affecting economic activities after kidney transplantation

⑥ Others

Q5. How long do you think the donor should rest after nephrectomy? Please reply based on the discharge date.

① 1-2 weeks ② 3-4 weeks

③ 5-8 weeks ④ 9-12 weeks ⑤ 13 weeks or more

***Opinions on policy support for live kidney donors***

Q6. Do you think the government should provide social and economic support to donors who donate their kidney?

① Yes → Go to Q6-1

② No → Go to Q7

③ I don't know → Go to Q7

Q6-1. What kind of policy support do you think the government should provide to donors who donated living kidneys? Please answer all.

① Support for donor nephrectomy and hospitalization expenses

② Additional support for kidney-related tests of national health examination after donor nephrectomy

③ Partial and full support of hospital expenses for follow-up monitoring of renal function after donor nephrectomy

④ Justification of sick leave within a certain period after donor nephrectomy

⑤ Support for salary during the leave of work

⑥ Others

Q7. Do you think the donor will be discriminated against or disadvantaged at work after living kidney donation?

① Yes → Go to Q7-1

② No → Go to the next page

③ I don't know → go to the next page

Q7-1. If you think you may face discrimination and disadvantages after donating a kidney, why? Please answer all applicable reasons.

① Because I think I will not be able to work during that period because I need a period of rest after donor nephrectomy

② Because I think that donating kidneys will weaken the physical function

③ Because smoking and drinking are not possible, also some diets are restricted, so I thought that it could cause discomfort in social life.

④ Other

***Presenting description of live kidney transplant***

**※ The following is a description of the contents of counseling about living kidney transplants that are actually being used in a hospital. Please read and understand the following before responding to the questionnaire later.**

| **1. Types of kidney transplant**  Kidney transplantations are largely divided into decreased donor kidney transplantation and live donor kidney transplantation. In Korea, more live donor kidney transplants are conducted due to the lack of deceased donors If there is no live kidney donor, end-stage kidney disease patients must wait for decreased donors, which will be registered with the Korean Network for Organ Sharing (KONOS) and received kidney transplants according to the strict selection criteria.  **2. Surgery and hospitalization / recovery period / post-discharge management**  The donor's left kidney is usually donated to the recipient. This is because the left kidney is more accessible, allowing for a laparoscopic nephrectomy. However, depending on various factors such as the donor's kidney condition or function, it may be changed to right nephrectomy with an open abdominal cavity. The operation time usually takes 2 to 4 hours, and it may vary depending on the condition of the kidney blood vessels or previous surgery history.  You will be discharged from the hospital 5 to 7 days after the surgery, and you will visit the outpatient clinic to remove the stitches about 1 week later. During one month after discharge, you need a rest for recovery, and if there are no special complications afterwards, your daily social life will not be affected. However, it is recommended to avoid excessive muscle exercise or physical work for up to 3 months, and we recommend that you receive a lifetime long regular follow-up for health care every year.  In accordance with the regulations on donor support for organs, sick leave and paid leave are recognized.   \| 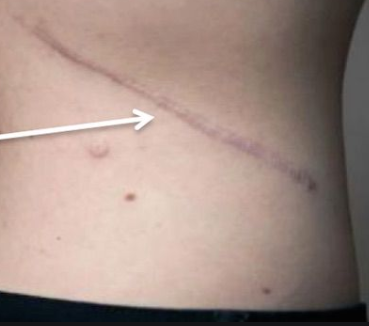 \| 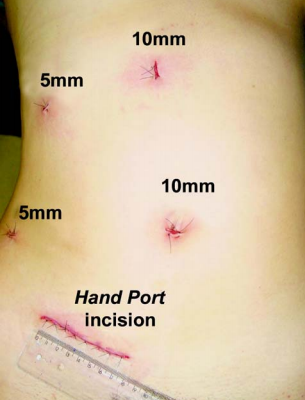 \| \| --- \| --- \| \| Examples of wounds  that occurred after open nephrectomy  ((https://juicing-for-health.com/prevent-kidney-stones-formation) \| Examples of wounds that occurred during laparoscopic nephrectomy  (Int Braz J Urol 31(5):421-430) \|   **3. Postoperative complications and side effects**  Donors may experience complications related to general anesthesia and nephrectomy. Examples include bleeding, pain, infection, pneumonia, embolism, renal failure, etc. The probability that a donor will dialysis after 10 years due to deterioration of kidney function is about 5 per 1,000 donors, and the mortality rate of kidney donors worldwide is 0.03%, which is not different from the mortality rate due to general anesthesia. |
| --- | --- | --- | --- | --- |

**※** We would like to ask you one more question after reading the description on a living donor kidney transplant. Please respond if you have changed your mind after reading the description.

***After reading the description on living donor kidney transplants: Awareness and perceptions of live donor kidney transplantation***

Q8. After reading the description on living donor kidney transplants, do you think you have a good understanding of kidney donation?

① Yes

② No

③ I don't know

Q8-1. If you have any additional questions about the descriptions for living kidney transplants you read earlier, please feel free to let us to know.

Q9. After reading the description on living donor kidney transplants, do you think a nephrectomy for living kidney donation is safe? Do you feel unsafe?

① Very safe

② It is a little safe

③ Normal

④ It’s not very safe

⑤ It’s not safe at all

Q10. After reading the description on living donor kidney transplants, how much do you think living kidney donation will affect your health in the long run? Do you think it won’t affect you?

① It will have a great impact

② It will have a little effect

③ Normal

④ It won't affect you very much

⑤ It will have no effect at all

***After reading the description on living donor kidney transplants: Willingness to donate kidney***

Q11. After reading the description on living donor kidney transplants, are you willing to donate your kidneys to a family member, relative, or other person?

① Willing to donate → Go to Q11-1

② I do not intend to donate → Go to Q11-3

⑨ I don't know → Go to Q12

Q11-1. If you donate your kidney, what relationship can you donate? Please rank up to 5th place. If you can donate only to the relationship that is ranked 1^st^, you only need to check the 1^st^ place. As another example, if donation are only available to parents and spouses, you need to rank up to the 2^nd^ priority.

① Parent

② Spouse

③ Brother/Sister

④ Son and daughter

⑤ Relatives within 4 villages

⑥ Relatives with 5 or more connections

⑦ Close friends and other relationships

⑧ others

Q11-2. Why are you willing to donate a kidney? Please answer all applicable reasons.

① For the recipient’s health

② For religious reasons

③ Saving a life feels rewarding

④ After donation, I thought that I could get any financial help from the recipient

⑤ I am convinced that there would be no other physical problems even after a kidney transplant

⑥ Others

→ After answering, go to Q5

Q11-3. If you are not willing to donate your kidneys, why? Please answer all applicable reasons.

① Because of vague fear due to lack of information

② Because it is difficult to access actual cases of kidney transplantation

③ Because of fear of possible medical complications after donation (

④ Because fear of effects on the kidneys and other organs after nephrectomy

⑤ Because of fear of affecting economic activities after kidney transplantation

⑥ Others

Q12. How long do you think the donor should rest after nephrectomy? Please reply based on the discharge date.

① 1-2 weeks

② 3-4 weeks

③ 5-8 weeks

④ 9-12 weeks

⑤ 13 weeks or more

***After reading the description on living donor kidney transplants: Opinions on policy support for live kidney donors***

Q13. Do you think the government should provide social and economic support to donors who donate their kidney?

① Yes → Go to Q13-1

② No → Go to Q14

③ I don't know → Go to Q14

Q13-1. What kind of policy support do you think the government should provide to donors who donated living kidneys? Please answer all.

① Support for donor nephrectomy and hospitalization expenses

② Additional support for kidney-related tests of national health examination after donor nephrectomy

③ Partial and full support of hospital expenses for follow-up monitoring of renal function after donor nephrectomy

④ Justification of sick leave within a certain period after donor nephrectomy

⑤ Support for salary during the leave of work

⑥ Others

Q14. Do you think the donor will be discriminated against or disadvantaged at work after living kidney donation?

① Yes → Go to Q14-1

② No → Go to Q15

③ I don't know → Go to Q15

Q14-1. If you think you may face discrimination and disadvantages after donating a kidney, why? Please answer all applicable reasons.

① Because I think I will not be able to work during that period because I need a period of rest after donor nephrectomy

② Because I think that donating kidneys will weaken the physical function

③ Because smoking and drinking are not possible, also some diets are restricted, so I thought that it could cause discomfort in social life.

④ Other

***Characteristics of participants***

Q1. Have you ever been married?

① Yes → Go to Q1-1

② No → Go to Q2

Q1-1. If you have ever been married, which of the following apply to you?

① Have a spouse and live together

② There is a spouse, but not living together (except for temporary conditions such as a business trip)

③ No spouse due to death of spouse

④ No spouse due to divorce

⑤ Don't know

Q2. What is your religion?

① Buddhism → Go to Q3

② Christianity → Go to Q3

③ Catholicism → Go to Q3

④ Confucianism → Go to Q3

⑤ Won Buddhism → Go to Q3

⑥ Others → Go to Q3

⑦ None → Go to Q4

Q3. To what extent do you dedicate in religious life?

① Dedicate very hard

② Dedicate hard

③ Just so-so

④ Not dedicated well

Q4. Please tell us your total number of brothers and sisters.

① No brothers/sisters

② 1 person

③ 2 people

④ 3 or more

Q5. Do you have any of the following disease? Please answer all of the following medical conditions you have.

① Diabetes

② high blood pressure

③ Chronic kidney disease

④ There is no applicable disease

Q6. Are there any patients in your family diagnosed with chronic kidney disease or end-stage kidney disease?

① Yes ② No ③ I don't know

Q7. Did you or any of your family members have received an organ or hematopoietic stem cell transplant?

① Yes → Go to Q7-1

② No → Go to Q8

③ I don't know → Go to Q8

Q7-1. What if you or any of your family members have had a transplant? Please respond to all applicable experiences.

① Living donor kidney transplant ② Deceased donor kidney transplant ③ Living liver transplant

④ Deceased donor liver transplant ⑤ Living donor lung transplant ⑥ Deceased donor lung transplant

⑦ Heart transplant ⑧ Pancreas transplant ⑨ Corneal transplant

⑩ Hematopoietic stem cell (bone marrow) transplant ⑪ Other transplants

Q8. Which of the following types of National Health Insurance do you have?

① National Health Insurance (Community)

② National Health Insurance (Workplace)

③ Full-aided

④ Partial-aided

⑤ Unsubscribed

⑥ Don't know

Q9. What is your job?

① Agriculture/forestry/fishing

② Self-employment

③ Sales and service

④ Production and labor

⑤ Management/professional

⑥ Housewives

⑦ Students

⑧ Unemployed/retired/others

Q10. How much is your monthly income? Please sum up your family’s income, such as bonuses, interest, and rent, as a monthly average.

① Less than $400 ② $400 ~ < $800 ③ $800 ~ < $1,200

④ $1,200 ~ < $1,600 ⑤ $1,600 ~ < $2,400 ⑥ $2,400 ~ < $3,200

⑦ $3,200 ~ < $4,800 ⑧ More than $4,800 ⑨ I don't know
