# Supplementary figures and images for: Perception regarding live kidney donation in the general population of South Korea
Source: PLoS One. 2022 Aug 4;17(8):e0272495. doi: 10.1371/journal.pone.0272495 (PMC9352025; doi:10.1371/journal.pone.0272495)

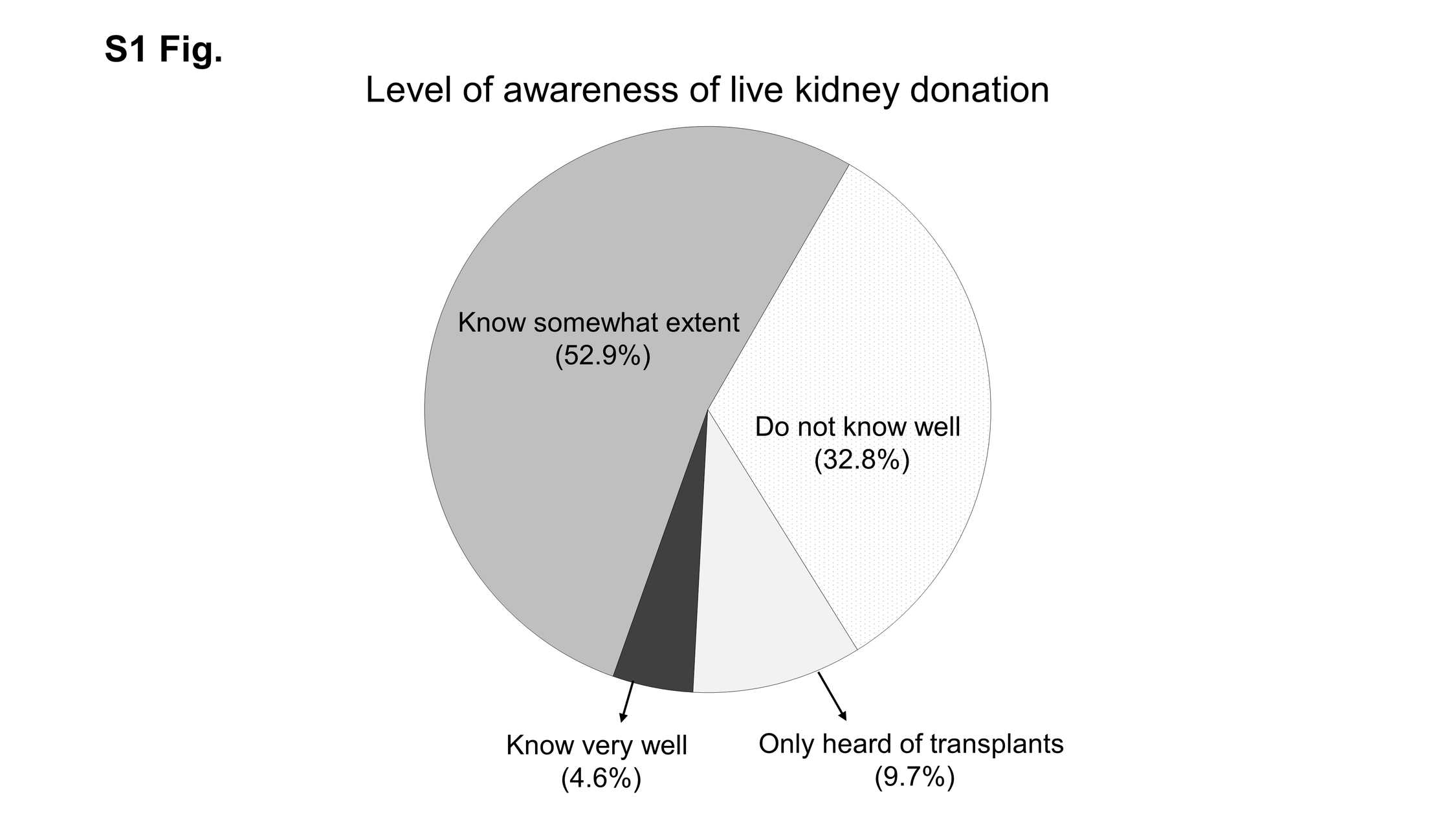

Supplement: S1 Fig — (TIF) [file pone.0272495.s002.tif]

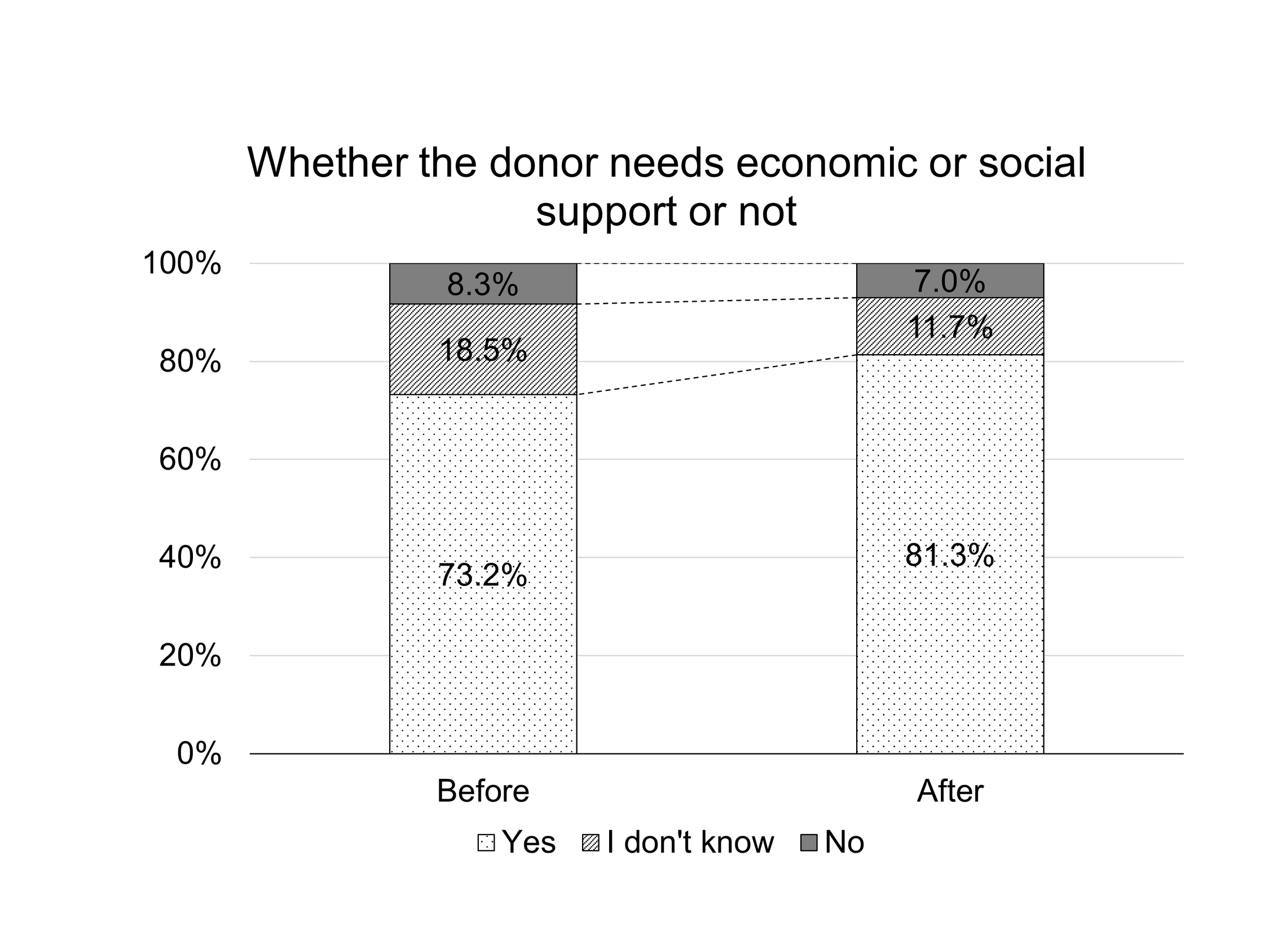

Supplement: S2 Fig — (TIF) [file pone.0272495.s003.tif]

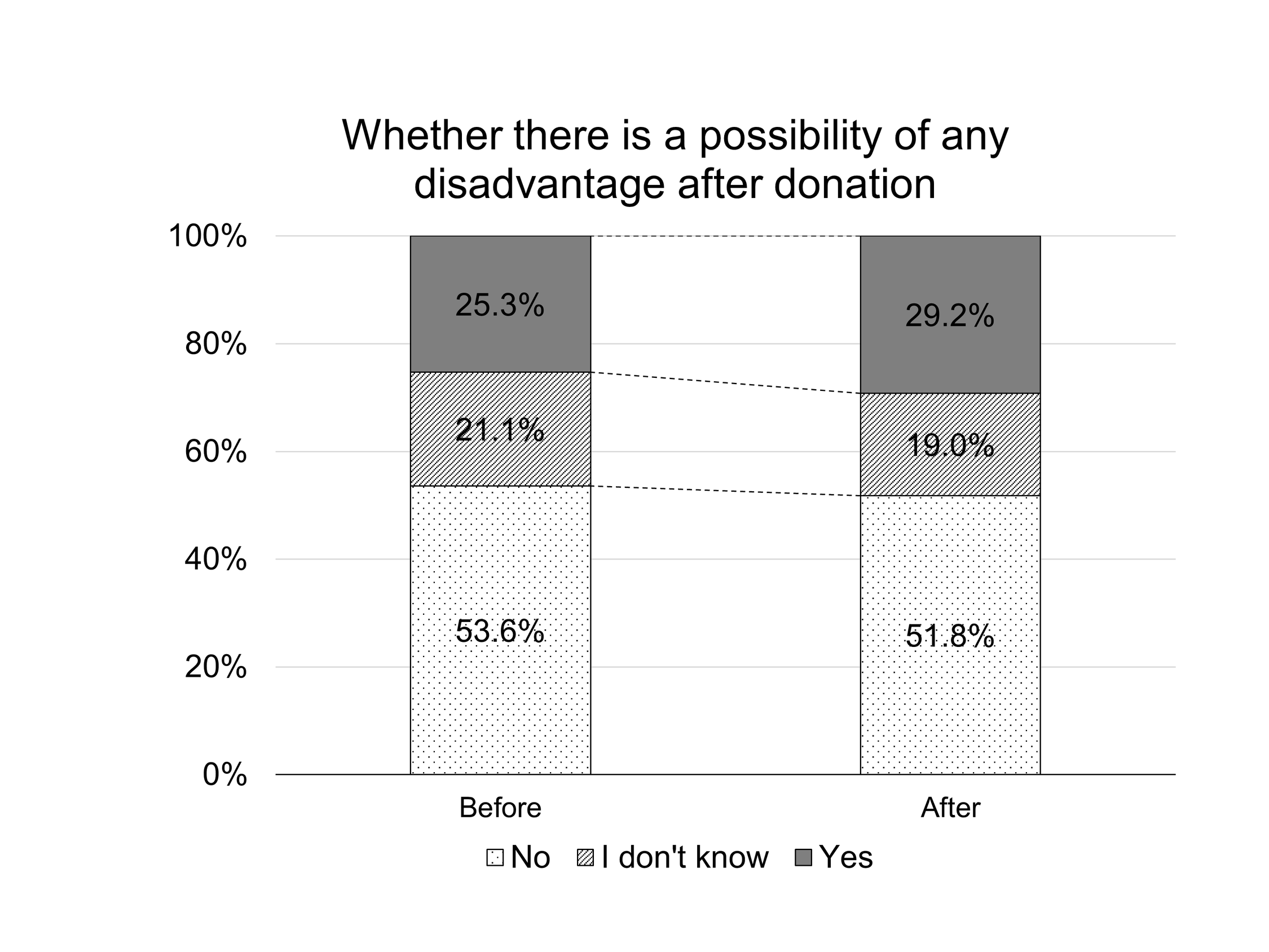

Supplement: S3 Fig — (TIF) [file pone.0272495.s004.tif]
