# Supplementary material for: Perception regarding live kidney donation in the general population of South Korea
Source: PLoS One. 2022 Aug 4;17(8):e0272495. doi: 10.1371/journal.pone.0272495 (PMC9352025; doi:10.1371/journal.pone.0272495)
Supplement: Attachment — Submitted filename: Response to Reviewers_Final.docx [file pone.0272495.s007.docx]

# Authors’ Response to the Review Comments

***Journal:***	PLOS ONE

***Manuscript #:***	PONE-D-22-06811

***Title of Paper:***	Perception regarding live kidney donation in the general population of South Korea

***Authors:***	Eunjeong Kang, Jangwook Lee, Sehoon Park, Yaerim Kim, Hyo Jeong Kim, Yong Chul Kim, Dong Ki Kim, Kwon Wook Joo, Yon Su Kim, Insun Choi, Hajeong Lee

We appreciate the time and efforts by the editor and referees for careful review of the manuscript and their valuable advice. Their review opinions were very helpful for making our manuscript more perfect. We have addressed all the issues indicated in the review report, and believe that the revised version can meet the journal publication requirements.

Response to Comments from Reviewer 1

**Comment 1:**

*Line 52: “…ranking second out of 70 countries in South Korea.” This is obviously an error. Is it Asia?*

## Response:

Thank you for your kind review comments regarding our manuscript. We apologize for the mistake in this sentence. According to the data released from International Registry in Organ Donation and Transplantation in 2020, South Korea has the second highest living donor transplant rate at 27.63 per million population (PMP) out of 70 countries. The figure of the quoted content is shown below:

Reference: <https://www.irodat.org/img/database/pdf/Irodat%20December_final%202020.pdf>)

**Comment 2:**

*Line 53: “commitment “would be a more appropriate word than “devotion” in this context.*

*Line 74: “even in many donors” should be “even among many donors”.*

*Lines 182-185: “When multiple choices were allowed, participants wanted to donate their kidneys in the order of their offspring (86.3%), spouse (85.5%), sibling (80.2%), parent (75.9%), a close friend (31.9%), relative (19.0%), and others (6.8%)”. It would be better read as “When multiple choices were allowed, participants wanted to donate their kidneys to their offspring (86.3%), spouse (85.5%), sibling (80.2%), parent (75.9%), a close friend (31.9%), relative (19.0%), and others (6.8%), in that order.”*

## Response:

We really appreciate this valuable comment. As the reviewer’s recommended, we corrected the words and sentences.

: “Since the devotion to giving a kidney to others” 🡪 “Since the commitment to giving a kidney to others”

: “even in many donors” 🡪 “even among many donors”

: “When multiple choices were allowed, participants wanted to donate their kidneys in the order of their offspring~” 🡪 “When multiple choices were allowed, participants wanted to donate their kidneys to their offspring~”

**Comment 3:**

*Lines 195 and 206: References in the text to the figures 3A and 3B are not clear. According the figures, it’s better to refer to the titles as “Reasons for willingness to a live-kidney donation” and “Reasons for reluctance to a live-kidney donation”.*

## Response:

We really appreciated your comment. We modified the title of fig 3A and 3B as you recommended.

**Comment 6:**

*Line 217: “leave of work” should be “leave from work”.*

## Response:

Thank you for your kind review and we revised as you recommended.

**Comment 7:**

*Line 251: This line gives the impression that deceased-donor kidney transplants (DDKT) are not done at all in Korea. But according to data, about 1500 DDKT were done in 2018.*

## Response:

We really appreciate this valuable comment. There is a possibility that the meaning of the sentence is conveyed incorrectly, so we tried to change it as follows:

“Additionally, the reasons why deceased donor transplantation has not been performed in Korea are as follows:” 🡪 “Additionally, the reason why deceased donor transplantation in Korea is less than that of living donors is as follows:”

**Comment 8:**

*Line 252: How can the high rate of deaths from head trauma be a reason for NOT doing DDKT?.*

## Response:

We appreciate the meticulous comment. We didn’t notice the wrong word choice in the process of citing the Korean research paper. We have modified the sentences as follows to convey the meaning better.

“1) the leading cause of brain death, such as death from head trauma caused by traffic or cerebrovascular accident, is high;” 🡪 “1) the leading causes of brain death, such as death from head trauma caused by traffic or cerebrovascular accident, have gradually decreased as the development of emergency treatment did not lead to death”.

**Comment 9:**

*Line 264 and 265: “Unlike in Western countries, living kidney donation was not encouraged in Korea because it was already performed based on a family-centered community culture in Korea”. This sentence is not clear. Please re-word it to give a proper meaning.*

## Response:

Thanks for the important comment. The sentence the reviewer mentioned was that there is no need to encourage live kidney donation because of the family-oriented culture in Korea. In other words, even if the nations or society does not encourage live organ transplantation, donating kidneys for the family is taken for granted in Korea. We revised the main manuscript as follows to clarifying the meaning:

“Unlike in Western countries, living kidney donation was not encouraged in Korea because it was already performed based on a family-centered community culture in Korea.”

🡪 “Unlike in Western countries, live kidney donation is being actively conducted even though live kidney transplant is not systemically encouraged, which may be due to the family-centered culture in Korea.”

**Comment 10:**

*Table 2: First Column: Title should be “Willing to donate”.*

## Response:

Thank you for your comment. The column title has been changed from “Willing to date” to “Willing to donate”.

**Comment 11:**

*There are two figs 2A. No Fig. 2B, although it’s referred to in the text.*

## Response:

We appreciated your valuable comments and apologize for the mistake. Figure 2A was uploaded in duplicate in the process of uploading files. This time, we uploaded correct figure 2B file.

Response to Comments from Reviewer 2

## Overall comment:

*This is a well written and very clear manuscript. The followings are my observations to improve the manuscript.*

## Comment 1:

*Key Words: Key words are not MeSH terms except Kidney transplantation. Please use MeSH terms*

## Response:

We appreciated your valuable comments. We combined the words because we thought there was a lack of MeSH term to properly express the subject of this study. As you recommended, we changed the keyword as below:

**“Keywords**: Kidney transplantation, Live kidney donations, Organ donor perceptions, Kidney transplant options”

🡪“**Keywords**: Kidney transplantation, organ donation, perceptions

## Comment 2:

*Study design*

*This study could have been planned as a qualitative study as a lot of information is restricted due to nature of the study design. Quantitative designing will not explore the exact picture of study in this nature.*

## Response:

Thank you for your kind review comments regarding our manuscript. We, of course, considered the implementation of qualitative research first when planning this study, however, we could not proceed considering resources and time. In addition, this study is likely to be the foundation for a change in Korea’s transplant-related policies, so we judged that it would be more appropriate to listen to the opinions of many individuals as possible. Thus, this questionnaire was conducted as a quantitative study. As you pointed out, we also think that further studies on this field should be carried out in the future.

## Comment 3:

## *Table 2*

## *- The first column – have a column title*

## *- The second column – Check the column title*

## *- P value – three decimal points may not be necessary. One or two decimal points would be adequate*

## *% - Indicating percentage mark at the end of each value may not be required. You may include a foot note that the values are expressed in number (%). Then readers know that the value in the brackets is the percentage.*

## Response:

Thank you for your kind review comments regarding our manuscript. All the points the reviewer pointed out regarding table 2 were reflected and revised as follows:

**Table 2. Characteristics according to willingness of kidney donation^a^**

| **Characteristics** | **Willing to donate** | **Do not intend to donate** | **Undetermined** | **Total** | **p** |
| --- | --- | --- | --- | --- | --- |
|  | **(N=511)** | **(N=136)** | **(N=353)** | **(N=1000)** |  |
| **Marital status** |  |  |  |  | 0.75 |
| Married | 370 (72.4) | 94 (69.1) | 254 (72.0) | 718 (71.8) |  |
| Unmarried | 141 (27.6) | 42 (30.9) | 99 (28.0) | 282 (28.2) |  |
| **Religion** |  |  |  |  | 0.13 |
| Buddhism | 78 (15.3) | 19 (14.0) | 62 (17.6) | 159 (15.9) |  |
| Christianity | 134 (26.2) | 34 (25.0) | 78 (22.1) | 246 (24.6) |  |
| Christianity | 65 (12.7) | 13 (9.6) | 41 (11.6) | 119 (11.9) |  |
| Others | 1 (0.2) | 2 (1.5) | 6 (1.7) | 9 (0.9) |  |
| None | 233 (45.6) | 68 (50.0) | 166 (47.0) | 467 (46.7) |  |
| **Dedication in religious life** |  |  |  |  | 0.66 |
| Dedicate very hard | 29 (10.4) | 7 (10.3) | 16 (8.6) | 52 (9.8) |  |
| Dedicate hard | 101 (36.3) | 23 (33.8) | 60 (32.1) | 184 (34.5) |  |
| Pretty well | 98 (35.3) | 20 (29.4) | 70 (37.4) | 188 (35.3) |  |
| Not dedicated well | 50 (18.0) | 18 (26.5) | 41 (21.9) | 109 (20.5) |  |
| **Total number of brothers and sisters** |  |  |  |  | 0.52 |
| No brothers/sisters | 27 (5.3) | 5 (3.7) | 16 (4.5) | 48 (4.8) |  |
| 1 | 139 (27.2) | 37 (27.2) | 87 (24.6) | 263 (26.3) |  |
| 2 | 105 (20.5) | 38 (27.9) | 87 (24.6) | 230 (23.0) |  |
| 3 or more | 240 (47.0) | 56 (41.2) | 163 (46.2) | 459 (45.9) |  |
| **Chronic disease status** |  |  |  |  | 0.77 |
| Diabetes | 53 (10.4) | 14 (10.3) | 29 (8.2) | 96 (9.6) |  |
| Hypertension | 99 (19.4) | 23 (16.9) | 61 (17.3) | 183 (18.3) |  |
| Chronic kidney disease | 3 (0.6) | 2 (1.5) | 3 (0.8) | 8 (0.8) |  |
| No chronic disease | 356 (69.7) | 97 (71.3) | 260 (73.7) | 713 (71.3) |  |
| **Family history of chronic or end-stage kidney disease** |  |  |  |  | 0.55 |
| Yes | 34 (6.7) | 8 (5.9) | 16 (4.5) | 58 (5.8) |  |
| No | 423 (82.8) | 118 (86.8) | 302 (85.6) | 843 (84.3) |  |
| Unknown | 54 (10.6) | 10 (7.4) | 35 (9.9) | 99 (9.9) |  |
| **Health insurance enrollment types** |  |  |  |  | 0.7 |
| National Health Insurance (Community) | 138 (27.0) | 41 (30.1) | 101 (28.6) | 280 (28.0) |  |
| National Health Insurance (Workplace) | 324 (63.4) | 83 (61.0) | 211 (59.8) | 618 (61.8) |  |
| Full-aided | 12 (2.3) | 1 (0.7) | 6 (1.7) | 19 (1.9) |  |
| Partial-aided | 4 (0.8) | 0 (0.0) | 2 (0.6) | 6 (0.6) |  |
| Unsubscribed | 6 (1.2) | 2 (1.5) | 3 (0.8) | 11 (1.1) |  |
| Unknown | 27 (5.3) | 9 (6.6) | 30 (8.5) | 66 (6.6) |  |
| **Job** |  |  |  |  | 0.23 |
| Agriculture/forestry/fishing | 6 (1.2) | 1 (0.7) | 4 (1.1) | 11 (1.1) |  |
| Self-employment | 32 (6.3) | 13 (9.6) | 14 (4.0) | 59 (5.9) |  |
| Sales and service | 53 (10.4) | 10 (7.4) | 28 (7.9) | 91 (9.1) |  |
| Production and labor | 49 (9.6) | 10 (7.4) | 39 (11.0) | 98 (9.8) |  |
| Management/professional | 178 (34.8) | 52 (38.2) | 125 (35.4) | 355 (35.5) |  |
| Housewives | 70 (13.7) | 21 (15.4) | 66 (18.7) | 157 (15.7) |  |
| Students | 34 (6.7) | 12 (8.8) | 16 (4.5) | 62 (6.2) |  |
| Unemployed/retired/others | 89 (17.4) | 17 (12.5) | 61 (17.3) | 167 (16.7) |  |
| **Household monthly income** |  |  |  |  | 0.47 |
| Less than $1,600 | 94 (18.4) | 30 (22.1) | 69 (19.5) | 193 (19.3) |  |
| $1,600 to < $3,200 | 205 (40.1) | 52 (38.2) | 140 (39.7) | 397 (39.7) |  |
| $3,200 to $4,800 | 128 (25.0) | 21 (15.4) | 78 (22.1) | 227 (22.7) |  |
| More than $4,800 | 68 (13.3) | 30 (22.1) | 53 (15.0) | 151 (15.1) |  |
| Unknown | 16 (3.1) | 3 (2.2) | 13 (3.7) | 32 (3.2) |  |

^a^The values are expressed in number (%)

## Comment 4:

*Figures*

*Many figures are a repetition of the content in the body. If the figure does not explain anything in addition to the content in the body, such figures can be taken out*

## Response:

We really appreciate this valuable comment. As the reviewer’s recommended, Fig 1 and Fig 4 has been moved to supporting information part.
