# Supplementary material for: Perception regarding live kidney donation in the general population of South Korea
Source: PLoS One. 2022 Aug 4;17(8):e0272495. doi: 10.1371/journal.pone.0272495 (PMC9352025; doi:10.1371/journal.pone.0272495)
Supplement: Attachment — Submitted filename: Reviewer comments.pdf [file pone.0272495.s006.pdf]

This is a well written and very clear manuscript. The followings are my observations to improve the manuscript.

Key Words: Key words are not MeSH terms except Kidney transplantation. Please use MeSH terms

Study design

This study could have been planned as a qualitative study as a lot of information is restricted due to nature of the study design. Quantitative designing will not explore the exact picture of study in this nature.

Table 2

The first column – have a column title

the second column – Check the column title

P value – three decimal points may not be nessasary. One or two decimal points would be adequate

% - Indicating percentage mark at the end of each value may not be required. You may include a foot note that the values are expressed in number (%). Then readers know that the value in the brackets is the percentage.

Figures

Many figures are a repetition of the content in the body. If the figure does not explain anything in addition to the content in the body, such figures can be taken out
